# Dissociative Model in Patients With Resistant Schizophrenia

**DOI:** 10.3389/fpsyt.2022.845493

**Published:** 2022-02-15

**Authors:** Georgi Panov

**Affiliations:** Psychiatric Clinic, University Hospital for Active Treatment “Prof. D-R Stoian Kirkovic”, Trakia University, Stara Zagora, Bulgaria

**Keywords:** resistance, schizophrenia, dissociation, resistant schizophrenia, treatment, diagnosis, antipsychotic drugs

## Abstract

**Background:**

Schizophrenia is a severe mental illness in which, despite the growing number of antipsychotics from 30 to 50% of patients remain resistant to treatment. Many resistance factors have been identified. Dissociation as a clinical phenomenon is associated with a loss of integrity between memories and perceptions of reality. Dissociative symptoms have also been found in patients with schizophrenia of varying severity. The established dispersion of the degree of dissociation in patients with schizophrenia gave us reason to look for the connection between the degree of dissociation and resistance to therapy.

**Methods:**

The type of study is correlation analysis. 106 patients with schizophrenia were evaluated. Of these, 45 with resistant schizophrenia and 60 with clinical remission. The Positive and Negative Syndrome Scale (PANSS) and Brief Psychiatric Rating Scale (BPRS) scales were used to assess clinical symptoms. The assessment of dissociative symptoms was made with the scale for dissociative experiences (DES). Statistical methods were used to analyze the differences in results between the two groups of patients.

**Results:**

Patients with resistant schizophrenia have a higher level of dissociation than patients in remission. This difference is significant and demonstrative with more than twice the level of dissociation in patients with resistant schizophrenia.

The level of dissociation measured in patients with resistant schizophrenia is as high as the points on the DES in dissociative personality disorder.

**Conclusion:**

Patients with resistant schizophrenia have a much higher level of dissociation than patients in clinical remission. The established difference between the two groups support to assume that resistance to the administered antipsychotics is associated with the presence of high dissociation in the group of resistant patients. These results give us explanation to think about therapeutic options outside the field of antipsychotic drugs as well as to consider different strategies earlier in the diagnostic process.

## Background

Schizophrenia is a serious mental illness which is characterized by changes in information processing as a consequence of misinterpretation of stimuli from the external environment. As a result, the clinical picture is characterized by positive symptoms (delusions and hallucinations), negative (apathy, anhedonia, dull affect, and loss of social cohesion), and cognitive ones with changes in attention and working memory. In addition to these clinically important symptoms for the diagnosis of schizophrenia, depressive, anxious, and cognitive symptoms are common if not always present (1). These psychiatric manifestations are associated with metabolic, lipid, and immune changes, often requiring additional therapeutic approaches (2, 3). Interesting observations on the level of serum lipids have been made in patients with schizophrenia and in persons using psychostimulants. Decreases in serum lipid levels were observed in both groups (3). Studies assessing self-perception and assessment of interpersonal space have been performed (4). There is evidence that as anxiety increases, interpersonal space increases (5). Such analyzes have also been performed in patients with schizophrenia who show that they have an increase in interpersonal space (6). Impaired cognitive assessment of reality and self-perception is associated with changes in behavior and the appearance of typical symptoms of schizophrenia associated with metabolic disorders as well as changes in neural connections between brain regions (7). This complex picture of changes in perception, behavior, metabolic characteristics, and functional connectivity makes schizophrenia a therapeutic challenge.

This is the reason despite the constant expansion of various therapeutic interventions, a significant percentage of patients remain resistant and pose a serious personal family and social problem. Some authors try to consider patients with resistant schizophrenia as a separate category. This raises the question of looking for different therapeutic approaches in them (2, 8, 9).

Janet presented the concept of dissociation for the first time at the end from 1,800, which is defined as a failure to integrate experiences that are usually related to each other in stream of consciousness (10). Dissociation is the partial or complete loss of normal integration between memories of the past, awareness of one's identity and immediate sensations, and control of bodily movements (11). Dissociation is a special form of consciousness in which events that would normally be related are separated from each other (12). Some authors (13) believe that dissociation is not only pathological, but may also play a role in some adaptive functions. It is also observed in healthy individuals in certain conditions (14).

Historically, dissociation as a clinical phenomenon has been associated with the presence of traumatic events leading to dissociative symptoms (15). A link has been found between dissociative symptoms and traumatic childhood events. Putnam (13) found that the most important traumas originate from childhood due to physical or sexual abuse with subsequent development of symptoms often after many years. According to the same author, dissociative symptoms also often occur in adults with severe traumatic event or a series of traumatic events. He found this in about half of the cases of dissociation. On the other hand, in direct clinical practice with adult patients, it is difficult to make a retrospective assessment of childhood experiences in order to give them the appropriate clinical weight. The authors found that 59.6% of 468 patients with a proven history of childhood sexual abuse were unable to recall episodes of past violence (16). Contradictory data are also available. The problem with the analysis of trauma in early childhood in the evaluation of adult patients is related to the fact that the manifestation of false memory experiences for the presence of trauma is often provoked (17).

It was found a traumagenic neurodevelopmental (TN) model of schizophrenia. Authors find the similarities between the effects of traumatic events on the developing brain and the biological abnormalities found in persons diagnosed with schizophrenia (12). The current diathesis-stress model of schizophrenia proposes that a genetic deficit creates a predisposing vulnerability in the form of oversenstivity to stress (15).

Corresponding changes in interpersonal space and self-esteem have been found in patients with dissociative disorders as well as in patients with schizophrenia (5, 18). Low levels of serum lipids have been reported (19) as well as impaired functional connectivity between brain regions (20).

The connection between dissociation and psychosis has been examined by Eugen Bleuler in patients with schizophrenia (21). In his Textbook of Psychiatry, he writes (21): “It is not only in hysteria that one finds an arrangement of different personalities who inherit from each other. Through such a mechanism, schizophrenia gives rise to different personalities existing side by side [(21), p. 138]. Bleuler has suggested that schizophrenia is a division of mental relationships similar to hysteria, but in a very extreme form. Psychotic decompensation of some individuals with psychotic symptoms, such as hallucinations, may occur. There are also a large number of observations showing a high level of dissociation in patients with schizophrenia (15, 22–27). The above data suggest a close link between schizophrenia and dissociative disorders. Several studies have found surprisingly high coincidences in the symptoms of these diagnoses (27–30). Even the symptoms described by Kurt Schneider as pathognomonic for schizophrenia have been proposed to be more characteristic of dissociative disorders (31, 32). Other studies suggest that there are similarities only in hallucinatory production as a characteristic of voices and their expression, but not in the presence of formal thought disorders, bizarre delusions, and negative symptoms (33). Studies indicate that up to 50% of patients with psychosis have severe dissociative symptoms (34, 35). This established overlap of symptoms in schizophrenia and dissociative disorder raises questions about therapy and expectations that a similar pattern of response will be observed. The data show results contrary to expectations. On the one hand, the treatment of schizophrenia is mainly with antipsychotic drugs, and on the other hand, the treatment of dissociative phenomena with antipsychotic drugs is generally ineffective (36). Examining the relationship between schizophrenia and dissociation, some authors raise the question of the existence of a subtype of schizophrenia, allowing for a new conceptualization of the relationship between them (37).

Resistance to drug therapy is registered in about 30–50% of patients with schizophrenia (38–44). The analysis of the relationship between dissociation in patients with schizophrenia and the course of the disease in them shows that those with a high degree of dissociation have a more severe course and more pronounced symptoms (45).

In the analysis of the literature available to us, we did not find a comparative study of the differences in dissociative symptoms in patients with resistant schizophrenia and those in clinical remission.

Working hypothesis: We suppose that the level of dissociation in patients with resistance to therapy will be higher than those in clinical remission.

## Materials and Methods

105 patients with schizophrenia were observed. Of these, 45 have resistant **s**chizophrenia and the remaining 60 are in clinical remission.

Including criteria for patients with resistant schizophrenia are those who have met the resistance criteria of the published consensus on resistant schizophrenia (46). They are:

1. Assessment of symptoms with the Positive and Negative Syndrome Scale (PANSS) and Brief Psychiatric Rating Scale (BPRS) scale (47, 48).
2. Prospective monitoring for a period of at least 12 weeks.
3. Administration of at least two antipsychotic medication trials at a dose corresponding to or greater than 600 mg chlorpromazine equivalents.
4. Reduction of symptoms when assessed with the PANSS and BPRS scale by less than 20% for the observed period of time.
5. The assessment of social dysfunction using the SOFAS scale is below 60.

The exclusion criteria are:

1. Mental retardation
2. Presence of organic brain damage
3. Concomitant progressive neurological or severe somatic diseases.
4. Expressed personality change
5. Score of MMSI below 25 points.

The Dissociative Experiences Scale (DES) was used to assess dissociative symptoms (22).

The statistical software package SPSS, was used for statistical data processing.

Descriptive analyzes, correlation analysis, dispersion analysis, ANOVA, and a non-parametric statistical method were used [Mann Whitney U-test, (49)].

## Results

The mean age of patients in the group of resistant schizophrenia was 36.98 years. The minimum age is 21 years and the maximum is 60 years.

The mean age of patients in the group of schizophrenia in clinical remission was 37.25 years. The minimum is 23 years and the maximum is 63 years.

We do not find a difference in the mean age of the patients in the both groups at the time of the study.

The mean value of the dissociative symptoms scale found in all patients with schizophrenia was 29.1356, standard deviation was 22.3898, and the lowest and highest values were 0 and 97, respectively.

The mean value of the measured points with the Carlson and Putnam scale in patients with resistant schizophrenia is 42.578, and the standard deviation is 20.8977. Their average level of dissociation is commensurate with the level needed to diagnose dissociative personality disorder according to the interpretation of the scale (requires values above 48 on the scale).

For patients in clinical remission, the mean was 15.907 and the standard deviation was 14.530. Their mean level of dissociation is commensurate with the level required to diagnose schizophrenia according to the interpretation of the scale (requires values of 15.4).

**Figure 1:**
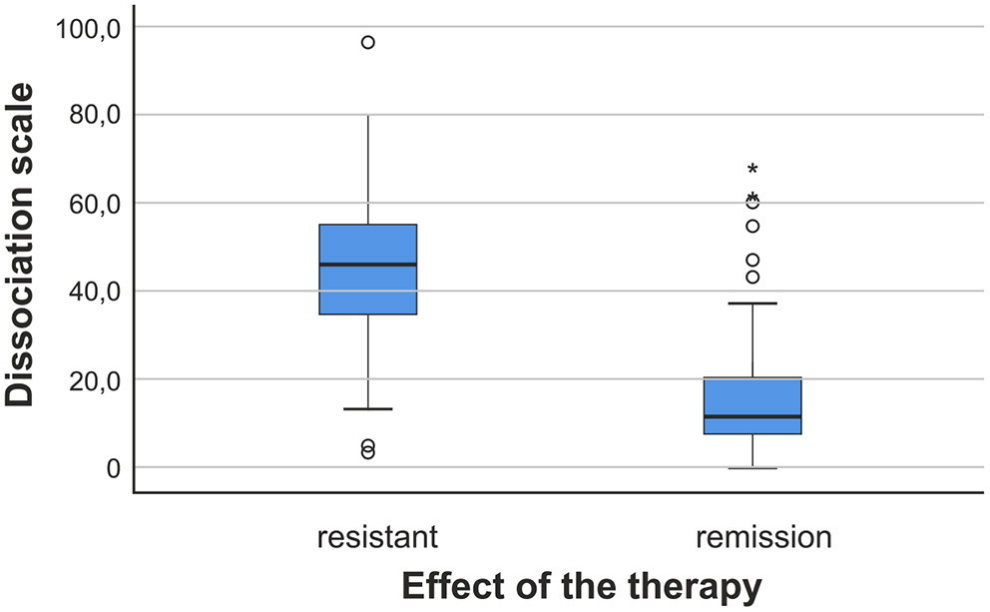
The variance of the results measured with DES in both groups of patients. The variance—in blue.

Up to three times the incidence of dissociation values is observed in patients with resistance compared to those in remission. The analysis of the median value in the two groups showed an even greater difference—up to four times higher in the group with patients resistant to therapy (Table 1; Figure 1).

**Table 1:** Descriptive analysis, the mean values, the median value, the standard deviation, and the standard error in the sample.

**Report**					
**Dissociation scale**					
**Effect of therapy**	**Mean**	***N***	**Std. deviation**	**Std. error of mean**	**Median**
Resistant	45.733	45	19.3313	2.8817	46.000
Remission	17.073	60	15.7153	2.0288	11.750
Total	29.356	105	22.3898	2.1850	22.500

In the analysis of the intragroup distribution of level of dissociation in patients with resistant schizophrenia, it was found that the main grouping of results is in the range of 30–60 on the scale used. This result shows that the majority of patients have a very high level of dissociation.

From the analysis of the distribution of data in patients in clinical remission, we found that the main distribution of patients is grouped in the range from 0 to about 20. We observe a much lower level of dissociation in patients in remission.

The statistical analysis of the results of Mann-Whitney U test is shown in Table 2 (Ranks) and Table 3 (Statistics) (^***^p < 0.001).

**Table 2:** Mann-Whitney U test—description.

**Ranks**				
	**Effect of therapy**	***N***	**Mean rank**	**Sum of ranks**
Dissociation scale	Resistant	45	75.18	3383.00
	Remission	60	36.37	2182.00
	Total	105		

**Table 3:** Mann-Whitney U test—statistics.

**Test Statistics**	
	**Dissociation scale**
Mann-Whitney U test	352.000
Wilcoxon W	2182.000
Z	−6.465
Asymp. Sig. (two -tailed)	0.000
Exact Sig. (two-tailed)	0.000
Exact Sig. (one-tailed)	0.000
Point probability	0.000

A variance analysis of the relationship between the level of dissociation and resistance to treatment is presented in Table 4.

**Table 4:** The variance analysis of the results of the two groups of patients.

**ANOVA Table**							
			**Sum of squares**	***df***	**Mean square**	***F***	**Sig**.
Dissociation scale Effect of therapy	Between groups	(Combined)	21121.601	1	21121.601	70.146	0.000
	Within groups		31014.037	103	301.107		
	Total		52135.638	104			

^***^p < 0.001.

The difference in the degree of dissociation registered by us in the two groups of patients raised the question: Is there a correlation between the value of dissociation and the values of the PANSS and BPRS scales.

The performed correlation analysis showed the presence of correlation *p* < 0.05, Table 5.

**Table 5:** The assessed mean values of the PANSS, BPRS, and DES scales.

	**Mean**	**Std. deviation**	***N***
PANSS positive	14.50	5.242	105
PANSS negative	16.95	6.316	105
PANSS disorganized	31.64	10.181	105
PANSS general	62.97	19.241	105
BPRS	45.12	13.266	105
DES	29.356	22.3892	105

Conducting a correlation analysis showed that there was a statistically significant correlation between the registered psychotic and dissociative symptoms (Table 6).

**Table 6:** Dispersion analysis between the dissociation scale (DES) and the clinical scales PANSS and BPRS.

**ANOVA Table**							
			**Sum of squares**	***df***	**Mean square**	***F***	**Sig**.
PANSS/Dissociation	Between groups	(Combined)	28544.064	58	492.139	2.274	0.002
	Within groups		9956.850	46	216.453		
	Total		38500.914	104			
BPRS/Dissociation	Between groups	(Combined)	12428.015	58	214.276	1.678	0.035
	Within groups		5875.375	46	127.726		
	Total		18303.390	104			

^*^p < 0.05.

## Discussion

Our results show a high degree of dissociation in patients with resistant schizophrenia, which is up to three times higher than in patients in clinical remission. We also find a correlation between the high values of the symptoms measured with the PANSS and BPRS scales and the dissociative symptoms registered with the DES.

Our study of dissociative symptoms coincides with the analysis of other teams, which show the presence of dissociative symptoms in patients with schizophrenia in the range from 11.9 to 44.24 (50–52). Some authors, in addition to assessing the dissociation, also make an analysis in dynamics: in admission and in stabilizing the condition. They do not get much change in the points on the DES from 19.2 to 14.1 (53). We find a mean score on dissociation level in all patients of 29.1356. Our data occupy an intermediate position compared to those described in the literature. Our results confirm the views of pioneers in schizophrenology such as Eugen Bleuler that schizophrenia is a state of extreme degree of dissociation (21).

In the previous studies, patients with resistance and those in clinical remission were not considered separately. The results of the points on the scale for dissociative experiences (DES) in patients in remission observed by us coincide with the criteria of the scale for patients with schizophrenia-−15.4. The results of other studies are mixed. We believe that this is because they have not considered patients separately—resistant and those in remission. Given that schizophrenia is a heterogeneous disease, it is also quite understandable the difference in the values of dissociative symptoms described in the individual studies. Over the years, there have been many analyzes of the overlap of symptoms of dissociative personality disorder and schizophrenia. Numerous studies have shown that up to 50% of patients with schizophrenia meet the diagnostic criteria for dissociative personality disorder (34, 35). These observations, as well as our data, do not show that in fact a probable reason for the lack of efficiency is the high degree of dissociation, which correlates positively with the high scores made with the PANSS and BPRS scales. This high level of resistance that we observe and register challenge of discussing the term resistance to treatment with “antidopaminergic drugs.” Dissociative disorders and symptoms have no effect from antipsychotic treatment (36).

In this sense, the question can be asked whether resistant schizophrenia is a form of dissociative disorder or mixture of the both entities. On the other hand, our results provide a basis for rethinking the diagnostic categories and research criteria used in the context of the relationship between the mind and the brain (54). The question remains whether high dissociation scales are the cause or consequence of the development of the neurodegenerative process in these patients (40, 55). Studies show that in some patients there is a progression of the disease, while in others there is a stationary condition that lasts for years. Magnetic resonance imaging data show that we can distinguish two groups of patients in comparative follow-up. Some have a neurodegenerative process and others do not (56).

The limitation of our study is related to the fact that we make a cross-section of the patient's condition in terms of dissociative symptoms. Longitudinal studies are needed to determine how the symptoms change over time. On the other hand, it is not clear whether the dissociative symptoms did not develop in parallel over time in patients with schizophrenia from the perspective of hospitalizations and in the process of treatment with various antipsychotic drugs. Some authors in a study found in the general population up to 1.7% of people at high risk of developing psychosis (57). No data have been established on the level of dissociative symptoms in them. Our study provides direction to consider assessment of dissociation early in the diagnostic process, especially in patients with the first psychotic episode, in order to discuss prognosis and associated therapy.

## Conclusion

We find a high degree of dissociation in patients with resistant schizophrenia. There is a high correlation between psychotic symptoms measured with the PANSS and BPRS scales and dissociative symptoms assessed with the DES. We found that the points on the scale for the level of dissociation in patients with resistant schizophrenia are as high as the requirement of the points on the scale for the assessment of dissociative personality disorder. The data we found for a high scale of dissociation in patients with resistance entitles us to seek therapeutic interventions outside the field of antipsychotic drugs. Therapeutic approaches in dissociative disorders may be considered (symptomatic and psychotherapeutic) as well as consideration of earlier use of electroconvulsive therapy (ECT) or transcranial magnetic stimulation (TMS).

## Data Availability Statement

The raw data supporting the conclusions of this article will be made available by the authors, without undue reservation.

## Ethics Statement

The studies involving human participants were reviewed and approved by Ethical committee of the University Hospital of Trakia University. The patients/participants provided their written informed consent to participate in this study.

## Author Contributions

The author confirms being the sole contributor of this work and has approved it for publication.

## Conflict of Interest

The author declares that the research was conducted in the absence of any commercial or financial relationships that could be construed as a potential conflict of interest.

## Publisher's Note

All claims expressed in this article are solely those of the authors and do not necessarily represent those of their affiliated organizations, or those of the publisher, the editors and the reviewers. Any product that may be evaluated in this article, or claim that may be made by its manufacturer, is not guaranteed or endorsed by the publisher.
